# Differences in perinatal and infant mortality in high-income countries: artifacts of birth registration or evidence of true differences?

**DOI:** 10.1186/s12887-015-0430-8

**Published:** 2015-09-04

**Authors:** Paromita Deb-Rinker, Juan Andrés León, Nicolas L. Gilbert, Jocelyn Rouleau, Anne-Marie Nybo Andersen, Ragnheiður I. Bjarnadóttir, Mika Gissler, Laust H. Mortensen, Rolv Skjærven, Stein Emil Vollset, Xun Zhang, Prakesh S. Shah, Reg S. Sauve, Michael S. Kramer, K. S. Joseph, for the Canadian Perinatal Surveillance System (Public Health Agency of Canada)

**Affiliations:** Maternal and Infant Health Section, Surveillance and Epidemiology Division, Centre for Chronic Disease Prevention, Public Health Agency of Canada, 785 Carling Avenue, AL 6804A, Ottawa, Ontario K1A 0K9 Canada; Department of Public Health, University of Copenhagen, Copenhagen, Denmark; Department of Obstetrics and Gynecology, Landspitali, Rekjavik, Iceland; National Institute of Health and Welfare (THL), Helsinki, Finland; Medical Birth Registry of Norway, University of Bergen, Bergen, Norway; Departments of Pediatrics and of Epidemiology, Biostatistics and Occupational Health, McGill University, Montreal, Quebec Canada; Department of Paediatrics, University of Toronto, Mount Sinai Hospital, Toronto, Ontario Canada; Departments of Pediatrics and Community Health Sciences, University of Calgary, Calgary, Alberta Canada; Department of Obstetrics and Gynaecology and the School of Population and Public Health, University of British Columbia, Vancouver, British Columbia Canada

**Keywords:** Birth registration, Infant mortality, Gestational age, Birth weight, Stillbirths, Neonatal mortality

## Abstract

**Background:**

Variation in birth registration criteria may compromise international comparisons of fetal and infant mortality. We examined the effect of birth registration practices on fetal and infant mortality rates to determine whether observed differences in perinatal and infant mortality rates were artifacts of birth registration or reflected true differences in health status.

**Methods:**

A retrospective population-based cohort study was done using data from Canada, United States, Denmark, Finland, Iceland, Norway, and Sweden from 1995–2005. Main outcome measures included live births by gestational age and birth weight; gestational age—and birth weight-specific stillbirth rates; neonatal, post-neonatal, and cause-specific infant mortality.

**Results:**

Proportion of live births <22 weeks varied substantially: Sweden (not reported), Iceland (0.00 %), Finland (0.001 %), Denmark (0.01 %), Norway (0.02 %), Canada (0.07 %) and United States (0.08 %). At 22–23 weeks, neonatal mortality rates were highest in Canada (892.2 per 1000 live births), Denmark (879.3) and Iceland (1000.0), moderately high in the United States (724.1), Finland (794.3) and Norway (739.0) and low in Sweden (561.2). Stillbirth:live birth ratios at 22–23 weeks were significantly lower in the United States (79.2 stillbirths per 100 live births) and Finland (90.8) than in Canada (112.1), Iceland (176.2) and Norway (173.9). Crude neonatal mortality rates were 83 % higher in Canada and 96 % higher in the United States than Finland. Neonatal mortality rates among live births ≥28 weeks were lower in Canada and United States compared with Finland. Post-neonatal mortality rates were higher in Canada and United States than in Nordic countries.

**Conclusions:**

Live birth frequencies and stillbirth and neonatal mortality patterns at the borderline of viability are likely due to differences in birth registration practices, although true differences in maternal, fetal and infant health cannot be ruled out. This study emphasises the need for further standardisations, in order to enhance the relevance of international comparisons of infant mortality.

**Electronic supplementary material:**

The online version of this article (doi:10.1186/s12887-015-0430-8) contains supplementary material, which is available to authorized users.

## Background

The infant mortality rate is a key population health indicator and commands widespread public attention since it reflects economic development, social equity, and health care services within a population [1]. It’s ready availability and importance as a health indicator makes the infant mortality rate a common tool for international comparisons of health status [2]. Several institutions like the United Nations Children’s Fund (UNICEF) and the Organization for Economic Cooperation and Development (OECD) use this and related indicators to rank countries annually [3, 4].

The popularity of the infant mortality indicator notwithstanding, international variations in birth registration laws and practices have the potential to bias comparisons of infant mortality. Problems can arise from differential registration of live births and stillbirths, especially births occurring at the borderline of viability (e.g., gestational age <22 weeks or a birth weight <500 grams, who typically do not survive the neonatal period), and/or their classification as stillbirths versus live births [5]. It is important to ascertain if international rankings by perinatal or infant mortality are affected by the above-mentioned biases, as such rankings often serve as the basis for evaluating national health care systems and for changing health policy. Although several studies [6–8] have suggested that variations in birth registration challenge international comparisons among high-income countries, the practice of annually ranking and comparing international infant mortality rates remains in vogue.

We examined variations in infant and fetal mortality rates based on birth registration practices at the borderline of viability, and the potentially differential classification of stillbirths and live births. We compared Canada and the United States to the Nordic countries because of the reported low levels of perinatal and infant mortality in the Nordic countries and their widely known emphasis on egalitarian ideals [9], lesser social disparities [10], and high quality of perinatal care [11].

## Methods

We used data for the period 1995 to 2005 from Canada, the United States and all five Nordic countries (Denmark, Finland, Iceland, Norway, and Sweden). We obtained reported counts of live births, stillbirths, neonatal deaths (0–27 days after birth) and post-neonatal deaths (28–364 days) by gestational age (GA) and birth weight (BW) from the vital registration systems or birth registries of each country. The gestational age categories that we examined included <22, 22–23, 24–25, 26–27, 28–31, 32–36, 37–41, ≥42 weeks and unknown gestational age, and birth weight categories included <500, 500–749, 750–999, 1000–1499, 1500–1999, ≥2000 grams and unknown birth weight. We also obtained reported counts of neonatal, post-neonatal, and infant deaths by cause of death (congenital anomalies, immaturity-related and other causes). In addition, we collected and contrasted the rules and registration requirements for stillbirths and live births for each country.

We obtained birth cohort linked live birth-infant death and fetal death data files for Canada from the vital registration files of Statistics Canada through an agreement between the Public Health Agency of Canada and Statistics Canada (which grants permission to the Agency to use this data). We excluded data from Ontario because of data quality concerns [12]. We obtained information from the United States from the birth cohort linked live birth-infant death data files and fetal death data files from the National Center for Health Statistics. Foreign residents and infants born at <20 weeks’ gestation in the United States were excluded from the study. We obtained Danish data from the Cause of Death Register and the Medical Birth Register of Denmark. The analyses were approved by the Danish Data Protection Agency. We obtained data for Finland from the Medical Birth Register of the National Institute for Health and Welfare (THL) and Cause-of-Death Register of Statistics Finland (no approval was needed). We obtained data for Iceland from the Landspitali University Hospital and participation in the study was approved by the Icelandic Directorate of Health. We obtained data for Norway from the Medical Birth Registry of Norway (MBRN) and the Cause of Death Registry. The data delivery was authorized by Medisinsk fødselsregisterforskriften. Data from Sweden was based on information from the Medical Birth Register and the Cause of Death Register and restricted to live births >22 weeks and stillbirths >28 weeks. Analyses of individual data were performed at Karolinska Institutet and permission was granted by the Ethical Committee of Karolinska Institutet.

Gestational age in Canada was based on the best clinical estimate of pregnancy duration as reported on the birth registration [12], while in the United States it was based on the last menstrual period substituted by the best clinical estimate of gestation (where available) if different from the menstrual estimate [13–15]. In Denmark, gestational age represented the clinical estimate made by the midwife at birth based on a combination of menstrual dates, menstrual cycle information and ultrasound scans (with approximately 70 % of women having received an ultrasound scan at 18 weeks gestation). In Sweden, gestational age was based on a hierarchical assessment, with 95 % of values based on early second trimester ultrasound and the remainder on menstrual dating or a pediatric assessment of the infant at birth [16]. Gestational age estimates in Finland, Iceland, and Norway were also based on a combination of information from menstrual, ultrasound and clinical dating.

We calculated the reported proportion of live births at each gestational age and birth weight category, and neonatal mortality rates by gestational age and birth weight. Similar analyses were carried out for stillbirth and post-neonatal mortality rates. Gestational age—and birth weight-specific stillbirth:live birth ratios (stillbirths per 100 live births) were also calculated to assess any proclivity towards labeling live births as stillbirths near the threshold of viability. Other comparisons included contrasts of cause of infant death, perinatal mortality and feto-infant mortality.

For all contrasts of interest, we calculated mortality rate ratios and 95 % confidence intervals to determine the magnitude and statistical significance of observed differences. We used Finland as the reference group for these calculations, as it had a reasonably large number of live births and mortality rates that were not outliers relative to other Nordic countries. We assessed the statistical significance of differences in rate ratios using 95 % confidence intervals and based on a test of heterogeneity of the odds ratios [17].The manuscript was prepared in line with the STROBE guidelines (see Additional file 1: STROBE Checklist). 

## Results

Gestational age and birth weight criteria for the registration of live births and stillbirths differed internationally. Countries such as Canada, the United States, Denmark, Finland, Iceland and Sweden required live birth registration for “any product of conception that shows signs of life at birth,” while Norway required registration of live births at a gestational age ≥16 weeks. Canada, Finland and Iceland required registration for stillbirths with a gestational age at delivery of ≥20 weeks or a birth weight ≥500 g; these criteria were ≥20 weeks or ≥350 g in the United States. The stillbirth registration criterion for fetal deaths was ≥16 weeks in Norway and ≥28 weeks in Sweden (until 2008), while Denmark changed its criterion from ≥28 weeks to ≥22 weeks in 2004. Canada, Denmark, Iceland and Sweden required stillbirth registration following medical pregnancy termination if birth weight and gestational age criteria for stillbirth were satisfied, whereas the United States, Finland and Norway required reporting of such cases as pregnancy terminations but not as stillbirths (Table 1).

**Table 1:** Gestational age and birth weight criteria for registration of live births and stillbirths among selected high-income countries, 1995–2005

Country	Live birth	Stillbirth	Stillbirth registration following termination of pregnancy^a^
Canada	Any signs of life regardless of gestational age or birth weight	≥20 weeks or ≥500 g	Yes
USA	Any signs of life regardless of gestational age or birth weight	≥20 weeks and/or ≥350 g^b^	No
Denmark	Any signs of life regardless of gestational age or birth weight	≥28 weeks till 2003 and ≥22 weeks from 2004	Yes
Finland	Any signs of life regardless of gestational age or birth weight	≥22 weeks or ≥500 g	No
Iceland	Any signs of life regardless of gestational age or birth weight	≥22 weeks or ≥500 g	Yes
Norway	≥16 weeks	≥16 weeks	No^c^
Sweden	Any signs of life regardless of gestational age or birth weight^e^	≥28 weeks	Yes

^a^If stillbirth definition is satisfied

^b^A few states in the US report fetal deaths for all periods of gestation

^c^In Norway, pregnancy terminations ≥16 weeks were reported as stillbirths prior to 1999 and reported on a special form since 1999 Sweden did not report stillbirths <28 weeks until 2008

^e^Data set provided by Swedish birth register only included live births from 154 days (22 completed weeks)

The proportions of live births <22 and 22–23 weeks were significantly higher in Canada and (especially) the United States as compared with the Nordic countries (Table 2). Differences between Canada and the Nordic countries were less evident at 24–25, 26–27 and 28–31 weeks gestation (with rates at 28–31 weeks being higher in Denmark and Norway than in Canada), although rates in the United States remained higher than in the other countries. Substantially higher proportions of live births with birth weights <500 g were reported in Canada and especially the United States compared with the Nordic countries. However, this pattern of variation was not observed among live births 750–999 g, 1000–1499 g and 1500–1999 g, with Canada (but not the United States) having rates similar to the Nordic countries (Table 2). The Nordic countries had higher rates of post-term births (≥42 weeks) compared with Canada and the United States.

**Table 2:** Distribution of live births by gestational age (GA) and birth weight (BW) among selected high-income countries, 1995–2005

Gestational age/Birth weight		Canada	USA	Denmark	Finland	Iceland	Norway	Sweden
		Number (%)	Number (%)	Number (%)	Number (%)	Number (%)	Number (%)	Number (%)
GA (weeks)	<22	1,494	33,691	64	6	0	101	not reported
		(0.07)	(0.08)	(0.01)	(0.001)	(0.00)	(0.02)	
	22–23	1,902	58,438	174	175	21	272	335
		(0.08)	(0.13)	(0.02)	(0.03)	(0.05)	(0.04)	(0.03)
	24–25	2,774	90,104	637	308	47	666	1,056
		(0.12)	(0.21)	(0.09)	(0.05)	(0.10)	(0.11)	(0.10)
	26–27	3,982	115,727	1,122	477	61	1,050	1,633
		(0.17)	(0.27)	(0.16)	(0.08)	(0.13)	(0.17)	(0.16)
	28–31	14,820	428,298	4,927	3,510	265	4,486	6,628
		(0.65)	(0.98)	(0.68)	(0.56)	(0.57)	(0.73)	(0.64)
	32–36	146,954	3,825,597	39,283	28,953	2,099	35,881	55,058
		(6.40)	(8.78)	(5.44)	(4.59)	(4.55)	(5.82)	(5.35)
	37–41	2,093,277	37,425,755	618,667	568,932	40,625	516,860	882,169
		(91.1)	(85.9)	(85.7)	(90.2)	(88.1)	(83.8)	(85.8)
	≥42	32,197	1,595,445	57,135	28,180	2,992	57,565	81,697
		(1.40)	(3.66)	(7.91)	(4.47)	(6.49)	(9.33)	(7.94)
	Unknown	5,699	432,844	6,642	5,369	199	25,258	1,273
		(0.25)	(0.98)	(0.91)	(0.84)	(0.43)	(3.93)	(0.12)
BW (grams)	<500	2,315	60,211	158	166	6	255	185
		(0.10)	(0.14)	(0.02)	(0.03)	(0.01)	(0.04)	(0.02)
	500–749	3,790	120,794	742	751	60	898	1,059
		(0.16)	(0.27)	(0.10)	(0.12)	(0.13)	(0.14)	(0.10)
	750–999	4,228	128,551	1,250	920	80	1,224	1,672
		(0.18)	(0.29)	(0.17)	(0.14)	(0.17)	(0.19)	(0.16)
	1,000–1,499	11,584	319,100	4,015	2,745	215	3,561	4,689
		(0.50)	(0.73)	(0.56)	(0.43)	(0.46)	(0.56)	(0.46)
	1,500–1,999	25,331	666,492	8,240	5,590	380	6,852	9,249
		(1.10)	(1.52)	(1.14)	(0.88)	(0.82)	(1.07)	(0.90)
	≥2000	2,250,300	42,694,623	707,730	624,694	45,563	628,658	1,008,332
		(97.9)	(97.1)	(98.0)	(98.4)	(98.4)	(98.0)	(98.4)
	Unknown	5,551	16,128	6,516	1,044	5	691	4,663
		(0.24)	(0.04)	(0.89)	(0.16)	(0.01)	(0.11)	(0.45)
Total		2,303,099 (100.0)	44,005,899 (100.0)	728,651 (100.0)	635,910 (100.0)	46,309 (100.0)	642,139 (100.0)	1,029,849 (100.0)

Percentages calculated after excluding live births with unknown gestational age or birth weight (except for total and unknown cells)

At 22–23 weeks gestation, Iceland had the highest neonatal mortality rate (1000.0 per 1000 live births); similar rates were observed in Canada and Denmark. Rates in Finland (794.3 per 1000 live births) were lower and similar to rates in the United States and Norway, while Sweden’s rates were significantly lower than in any other country (561.2 per 1000 live births, 95 % CI 506.2 to 615.1, *P <* 0.05). The mortality rate patterns were similar at 24–25 weeks between countries, but differed at later gestational ages. At 37–41 weeks gestation, neonatal mortality rates were lowest in Iceland, highest in Denmark and not significantly different among the other countries (Table 3). Patterns of birth weight-specific neonatal mortality were similar to those based on gestational age. Ratios of stillbirths to live births varied widely, with ratios in Canada lower than those in the United States, Finland and Norway at <22 weeks and <500 g. However, the United States had significantly lower stillbirth ratios than Canada, Finland, Iceland and Norway in the other preterm gestational age categories and in other birth weight categories <1,500 g (Table 4). Exclusion of births <28 weeks attenuated but did not eliminate the differences between Canada and the United States versus Finland.

**Table 3:** Neonatal mortality rates by gestational age (GA) and birth weight (BW) among selected high-income countries, 1995–2005

Gestational age/Birth weight		Canada	USA	Denmark	Finland	Iceland	Norway	Sweden
		Rate	Rate	Rate	Rate	Rate	Rate	Rate
		(95 % CI)	(95 % CI)	(95 % CI)	(95 % CI)	(95 % CI)	(95 % CI)	(95 % CI)
GA (weeks)	<22	950.5	850.9	875.0	1000.0	0.0	950.5	-^a^
		(938.2–960.9)	(847.0–854.6)	(768.5–944.5)	(540.7–1000.0)	(0.0–0.0)	(888.2–983.7)	
	22–23	892.2	724.1	879.3	794.3	1000.0	739.0	561.2
		(877.4–905.8)	(720.4–727.7)	(821.4–923.7)	(726.8–851.6)	(857.5–1000.0)	(682.5–790.1)	(506.2–615.1)
	24–25	417.4	311.7	456.8	318.2	340.4	304.8	269.9
		(399.0–436.1)	(308.6–314.7)	(417.6–496.4)	(266.5–373.4)	(208.6–493.1)	(270.0–341.3)	(243.3–297.8)
	26–27	141.9	118.1	181.8	136.3	131.1	106.7	140.2
		(131.2–153.1)	(116.3–120.0)	(159.7–205.7)	(106.8–170.4)	(58.4–242.2)	(88.6–126.9)	(123.7–158.0)
	28–31	44.4	36.5	43.8	46.2	34.0	38.1	42.2
		(41.1–47.8)	(35.9–37.1)	(38.3–49.9)	(39.5–53.6)	(15.6–63.5)	(32.7–44.1)	(37.5–47.4)
	32–36	7.0	5.9	7.9	8.5	7.6	6.9	8.6
		(6.6–7.4)	(5.9–6.0)	(7.0–8.8)	(7.4–9.6)	(4.4–12.3)	(6.0–7.8)	(7.8–9.4)
	37–41	0.9	0.9	1.3	0.9	0.5	1.0	0.8
		(0.9–0.9)	(0.9–0.9)	(1.2–1.4)	(0.8–0.9)	(0.3–0.8)	(0.9–1.0)	(0.7–0.9)
	≥42	1.3	1.1	1.6	1.1	0.7	1.1	1.2
		(0.9–1.7)	(1.0–1.1)	(1.3–1.9)	(0.7–1.5)	(0.1–2.4)	(0.8–1.4)	(1.0–1.5)
	Unknown	32.3	13.0	61.0	67.8	0.0	4.3	33.8
		(27.9–37.2)	(12.7–13.3)	(55.3–67.0)	(61.2–74.9)	(0.0–18.4)	(3.5–5.2)	(24.6–45.2)
BW (grams)	<500	914.0	838.3	905.1	795.2	833.3	760.8	578.4
		(901.9–925.1)	(835.4–841.3)	(848.3–945.9)	(725.7–853.8)	(358.8–995.8)	(703.6–811.8)	(503.7–650.5)
	500–749	533.8	420.8	481.1	472.7	633.3	368.6	330.5
		(517.7–549.8)	(418.1–423.6)	(444.6–517.8)	(436.5–509.1)	(499.0–754.1)	(337.0–401.1)	(302.2–359.7)
	750–999	163.9	118.4	175.2	162.0	150.0	111.1	112.4
		(152.9–175.4)	(116.7–120.2)	(154.5–197.4)	(138.7–187.4)	(80.0–247.4)	(94.0–130.1)	(97.7–128.6)
	1,000–1,499	53.4	42.4	61.0	51.4	18.6	45.5	52.2
		(49.4–57.7)	(41.7–43.1)	(53.8–68.9)	(43.2–60.6)	(5.1–46.9)	(38.9–52.9)	(46.1–59.0)
	1,500–1,999	19.7	18.3	22.5	23.8	10.5	16.2	21.5
		(18.1–21.5)	(18.0–18.6)	(19.4–25.9)	(20.0–28.1)	(2.9–26.7)	(13.3–19.5)	(18.7–24.7)
	≥2000	1.1	1.2	1.3	1.1	0.8	1.1	0.9
		(1.1–1.1)	(1.1–1.2)	(1.3–1.4)	(1.0–1.2)	(0.5–1.1)	(1.0–1.2)	(0.9–1.0)
	Unknown	36.4	135.4	66.0	12.5	0.0	94.1	55.8
		(31.6–41.7)	(130.2–140.8)	(60.1–72.3)	(6.6–21.2)	(0.0–521.8)	(73.3–118.3)	(49.3–62.7)
Total		3.8	4.4	3.5	2.5	2.1	2.6	2.2
		(3.7–3.8)	(4.4–4.4)	(3.3–3.6)	(2.4–2.6)	(1.7–2.6)	(2.5–2.8)	(2.1–2.3)

Rates expressed per 1,000 live births. ^a^Live births <22 weeks not recorded in the Swedish Medical Birth Register

**Table 4:** Stillbirth to live birth ratios, by gestational age (GA) and birth weight (BW) among selected high-income countries, 1995–2005

Gestational age/Birth weight		Canada	USA	Denmark	Finland	Iceland	Norway	Sweden
		Ratio (95 % CI)	Ratio (95 % CI)	Ratio (95 % CI)	Ratio (95 % CI)	Ratio (95 % CI)	Ratio (95 % CI)	Ratio (95 % CI)
GA (weeks)	<22	155.8 (145.9–166.4)	163.6 (161.4–165.9)	-^a^	200.0 (69.5–649.6)	-	2073.3 (1698.6–2559.6)	-^a^
	22–23	112.1 (105.3–119.3)	79.2 (78.3–80.2)	-	90.8 (72.8–113.3)	176.2 (100.5–316.8)	173.9 (149.5–202.6)	-
	24–25	38.2 (35.6–41.0)	26.7 (26.3–27.1)	-	37.0 (29.6–46.0)	42.6 (23.9–73.2)	40.7 (35.2–46.9)	-
	26–27	18.2 (16.8–19.7)	15.3 (15.0–15.5)	-	23.5 (18.9–28.9)	23.0 (11.9–41.5)	20.1 (17.2–23.3)	-
	28–31	9.6 (9.1–10.1)	7.6 (7.5–7.7)	11.4 (10.4–12.4)	9.4 (8.3–10.5)	10.9 (7.2–16.1)	8.6 (7.7–9.6)	9.2 (8.5–10.0)
	32–36	1.7 (1.6–1.7)	1.3 (1.3–1.3)	2.0 (1.9–2.2)	1.6 (1.5–1.7)	2.0 (1.4–2.7)	1.7 (1.6–1.9)	1.9 (1.8–2.0)
	37–41	0.2 (0.2–0.2)	0.1 (0.1–0.1)	0.2 (0.2–0.3)	0.1 (0.1–0.1)	0.1 (0.1–0.2)	0.2 (0.2–0.2)	0.2 (0.2–0.2)
	≥42	0.2 (0.1–0.2)	0.1 (0.1–0.1)	0.2 (0.2–0.2)	0.1 (0.0–0.1)	0.0 (0.0–0.2)	0.2 (0.2–0.2)	0.2 (0.2–0.2)
	Unknown	2.4 (2.1–2.9)	7.0 (7.0–7.1)	1.3 (1.1–1.6)	10.1 (9.2–11.0)	0.0 (0.0–1.9)	2.3 (2.1–2.5)	2.1 (1.4–3.1)
BW (grams)	<500	128.6 (121.7–135.8)	139.4 (137.9–140.8)	51.3 (38.7–67.4)	196.4 (162.4–238.2)	383.3 (151.8–1151.6)	1077.9 (947.1–1229.8)	13.5 (8.5–20.6)
	500–749	59.5 (56.4–62.7)	32.7 (32.3–33.1)	18.5 (15.3–22.2)	47.0 (41.3–53.4)	71.7 (47.3–107.8)	54.7 (48.9–61.1)	9.6 (7.8–11.8)
	750–999	22.0 (20.5–23.6)	13.8 (13.5–14.0)	11.4 (9.6–13.6)	21.1 (18.0–24.6)	21.3 (11.8–36.2)	16.8 (14.4–19.4)	8.6 (7.2–10.1)
	1,000–1,499	9.7 (9.1–10.3)	7.5 (7.4–7.6)	7.6 (6.7–8.5)	9.5 (8.3–10.8)	12.1 (7.7–18.2)	8.3 (7.3–9.3)	8.4 (7.6–9.3)
	1,500–1,999	4.2 (4.0–4.5)	3.3 (3.2–3.3)	3.8 (3.4–4.2)	4.3 (3.8–4.9)	2.4 (1.1–4.5)	3.8 (3.4–4.3)	4.5 (4.1–5.0)
	≥2000	0.2 (0.2–0.2)	0.2 (0.2–0.2)	0.3 (0.2–0.3)	0.2 (0.2–0.2)	0.2 (0.1–0.2)	0.2 (0.2–0.2)	0.2 (0.2–0.3)
	Unknown	15.8 (14.7–17.0)	308.0 (302.4–313.4)	7.3 (6.7–8.0)	4.7 (3.4–6.2)	0.0 (0.0–109.1)	48.0 (42.0–54.8)	2.5 (2.0–3.0)
Total		0.6 (0.6–0.6)	0.7 (0.7–0.7)	0.4 (0.4–0.5)	0.4 (0.4–0.4)	0.4 (0.4–0.5)	0.9 (0.9–0.9)	0.4 (0.3–0.4)

Ratios are expressed per 100 live births

^a^Denmark and Sweden did not report stillbirths <28 weeks until 2004 and 2008, respectively

Contrasts of crude neonatal mortality rates in 2005 showed that Canadian rates were 83 % (95 % CI 71–96) higher and U.S. rates were 96 % (95 % CI 93–99) higher than those in Finland (Table 5). Denmark had a significantly higher neonatal mortality rate (rate ratio 1.55) than Finland, while Sweden had a significantly lower rate (rate ratio 0.67). When live births <22 weeks gestation and live births <24 weeks were excluded from calculations of neonatal mortality, the differences between Canada and the United States versus Finland were substantially attenuated. Canadian neonatal mortality rates in 2005 among live births ≥28 weeks were 14 % (95 % CI 4–23) lower than rates in Finland, while the same rates in the United States were 9 % (95 % CI 7–11) lower than those in Finland. Neonatal mortality rates in Denmark and Sweden relative to Finland were not similarly affected by the exclusion of live births <28 weeks. Contrasts of neonatal mortality rates calculated after excluding live births <500 g and <1,000 g birth weight showed a similar pattern (Table 5).

**Table 5:** Crude neonatal mortality rates and rate ratios, and neonatal mortality rates and rate ratios after excluding live births <22 weeks, <24 weeks and <28 weeks, among selected high-income countries, 1995–2005 and 2005

Gestational age/Birth weight			1995-2005		2005	
			Rate (95 % CI)	Rate Ratio (95 % CI)	Rate (95 % CI)	Rate Ratio (95 % CI)
GA (weeks)	≥22	Canada	3.1 (3.1–3.2)	1.26 (1.23–1.29)	3.2 (2.9–3.4)	1.47 (1.36–1.59)
		USA	3.8 (3.7–3.8)	1.50 (1.49–1.51)	3.5 (3.5–3.6)	1.65 (1.62–1.67)
		Denmark	3.4 (3.3–3.5)	1.35 (1.30–1.41)	3.3 (2.8–3.7)	1.51 (1.32–1.73)
		Finland	2.5 (2.4–2.6)	1.00	2.2 (1.8–2.6)	1.00
		Iceland	2.1 (1.7–2.5)	0.83 (0.67–1.01)	1.4 (0.5–3.0)	0.65 (0.24–1.41)
		Norway	2.4 (2.3–2.5)	0.97 (0.92–1.02)	1.9 (1.5–2.3)	0.87 (0.72–1.06)
		Sweden	2.2 (2.1–2.3)	0.89 (0.86–0.93)	1.4 (1.2–1.7)	0.67 (0.57–0.79)
	≥24	Canada	2.4 (2.3–2.5)	1.05 (1.03–1.08)	2.4 (2.2–2.7)	1.24 (1.14–1.36)
		USA	2.8 (2.8–2.8)	1.22 (1.22–1.23)	2.6 (2.5–2.6)	1.32 (1.30–1.35)
		Denmark	3.2 (3.0–3.3)	1.39 (1.34–1.45)	3.0 (2.6–3.4)	1.53 (1.32–1.76)
		Finland	2.3 (2.2–2.4)	1.00	2.0 (1.6–2.4)	1.00
		Iceland	1.6 (1.2–2.0)	0.68 (0.53–0.86)	0.9 (0.3–2.4)	0.48 (0.13–1.22)
		Norway	2.1 (2.0–2.2)	0.92 (0.87–0.97)	1.5 (1.2–1.9)	0.79 (0.63–0.97)
		Sweden	2.0 (2.0–2.1)	0.90 (0.86–0.94)	1.4 (1.1–1.6)	0.70 (0.58–0.82)
	≥28	Canada	1.7 (1.6–1.7)	0.82 (0.79–0.85)	1.6 (1.4–1.7)	0.86 (0.77–0.96)
		USA	1.9 (1.8–1.9)	0.91 (0.91–0.92)	1.7 (1.6–1.7)	0.91 (0.89–0.93)
		Denmark	2.5 (2.4–2.6)	1.23 (1.18–1.29)	2.2 (1.9–2.6)	1.22 (1.03–1.43)
		Finland	2.0 (1.9–2.1)	1.00	1.8 (1.5–2.2)	1.00
		Iceland	1.0 (0.8–1.4)	0.51 (0.38–0.68)	0.5 (0.1–1.7)	0.26 (0.03–0.92)
		Norway	1.6 (1.5–1.7)	0.78 (0.73–0.83)	1.2 (1.0–1.6)	0.67 (0.52–0.85)
		Sweden	1.6 (1.5–1.6)	0.77 (0.73–0.81)	1.0 (0.8–1.2)	0.53 (0.43–0.65)
BW (grams)	≥500	Canada	2.8 (2.8–2.9)	1.23 (1.21–1.27)	2.7 (2.5–3.0)	1.40 (1.29–1.52)
		USA	3.3 (3.2–3.3)	1.42 (1.41–1.42)	3.0 (3.0–3.1)	1.53 (1.51–1.57)
		Denmark	3.3 (3.1–3.4)	1.42 (1.36–1.48)	3.1 (2.7–3.5)	1.56 (1.35–1.79)
		Finland	2.3 (2.2–2.4)	1.00	2.0 (1.6–2.4)	1.00
		Iceland	2.0 (1.6–2.5)	0.87 (0.70–1.07)	1.2 (0.4–2.7)	0.59 (0.19–1.39)
		Norway	2.2 (2.1–2.4)	0.97 (0.92–1.02)	1.7 (1.4–2.1)	0.87 (0.71–1.07)
		Sweden	2.1 (2.0–2.2)	0.92 (0.89–0.96)	1.4 (1.2–1.6)	0.71 (0.59–0.83)
	≥1000	Canada	1.7 (1.6–1.7)	1.10 (1.07–1.14)	1.4 (1.3–1.6)	1.04 (0.93–1.17)
		USA	1.8 (1.8–1.8)	1.17 (1.16–1.18)	1.6 (1.6–1.6)	1.18 (1.15–1.21)
		Denmark	2.5 (2.4–2.6)	1.64 (1.57–1.72)	2.3 (1.9–2.7)	1.67 (1.41–1.97)
		Finland	1.5 (1.4–1.6)	1.00	1.4 (1.1–1.7)	1.00
		Iceland	0.9 (0.7–1.3)	0.62 (0.45–0.83)	0.5 (0.1–1.7)	0.34 (0.04–1.24)
		Norway	1.5 (1.4–1.6)	1.00 (0.94–1.06)	1.2 (1.0–1.6)	0.92 (0.72–1.16)
		Sweden	1.6 (1.5–1.7)	1.06 (1.01–1.12)	1.0 (0.8–1.2)	0.75 (0.61–0.91)
All live births		Canada	3.8 (3.7–3.8)	1.50 (1.47–1.53)	3.9 (3.7–4.2)	1.83 (1.71–1.96)
		USA^a^	4.4 (4.4–4.4)	1.76 (1.75–1.76)	4.2 (4.2–4.3)	1.96 (1.93–1.99)
		Denmark	3.5 (3.3–3.6)	1.38 (1.33–1.43)	3.3 (2.9–3.8)	1.55 (1.35–1.77)
		Finland	2.5 (2.4–2.6)	1.00	2.2 (1.8–2.6)	1.00
		Iceland	2.1 (1.7–2.6)	0.84 (0.69–1.03)	1.4 (0.5–3.1)	0.65 (0.24–1.41)
		Norway	2.6 (2.5–2.8)	1.05 (1.00–1.10)	2.1 (1.7–2.5)	0.96 (0.79–1.15)
		Sweden	2.2 (2.1–2.3)	0.89 (0.85–0.93)	1.4 (1.2–1.7)	0.67 (0.57–0.79)

*GA* gestational age and *BW* birth weight. Rates are expressed per 1,000 infants at risk. Only births with gestational age <22 weeks excluded from the rate calculation for births **≥**22 weeks i.e., births with unknown gestational age included in the calculations. Calculations of rates for births **≥**28 weeks and birth weight **≥**500 g and **≥**1000 g were carried out in a similar manner. Finland was used as the reference category for calculation of rate ratios

^a^US rates are based on live births with GA ≥20 completed weeks

Between-country comparisons of crude stillbirth rates showed that Norway, the United States, and Canada had the highest rates (Appendix 1 Table 7). In 2005, Norway had a crude stillbirth rate that was 145 % (95 % CI 123–169) higher than Finland’s. Comparisons restricted to births ≥28 weeks rendered this difference non-significant (rate ratio 0.95, 95 % CI 0.80–1.12).

Table 6 shows crude, gestational age-specific and birth weight-specific post-neonatal mortality rates. Compared with Finland, crude rates of post-neonatal mortality in 2005 were 54 % (95 % CI 36–73) higher in Canada and 160 % (95 % CI 154–165) higher in the United States. Gestational age-specific comparisons after restriction to live births ≥22 weeks and ≥28 weeks did not significantly attenuate the differences between Canada and the United States versus Finland.

**Table 6:** Crude post-neonatal mortality rates and rate ratios, and post-neonatal mortality rates and rate ratios after excluding live births <22 weeks and <28 weeks, among selected high-income countries, 1995–2005 and 2005

Gestational age/Birth weight			1995-2005		2005	
			Rate (95 % CI)	Rate Ratio (95 % CI)	Rate (95 % CI)	Rate Ratio (95 % CI)
GA (weeks)	≥22	Canada	1.6 (1.5–1.6)	1.62 (1.57–1.68)	1.4 (1.2–1.5)	1.54 (1.36–1.73)
		USA	2.4 (2.3–2.4)	2.41 (2.40–2.43)	2.3 (2.3–2.3)	2.59 (2.54–2.65)
		Denmark	1.2 (1.1–1.3)	1.17 (1.09–1.25)	1.0 (0.8–1.3)	1.12 (0.86–1.43)
		Finland	1.0 (1.0–1.1)	1.00	0.9 (0.7–1.2)	1.00
		Norway	1.1 (1.1–1.2)	1.18 (1.09–1.26)	0.7 (0.5–1.0)	0.79 (0.56–1.08)
		Sweden	1.1 (1.0–1.2)	1.14 (1.07–1.21)	1.1 (0.9–1.3)	1.19 (0.98–1.44)
	≥28	Canada	1.5 (1.4–1.5)	1.49 (1.44–1.54)	1.2 (1.1–1.4)	1.43 (1.26–1.61)
		USA	2.1 (2.1–2.1)	2.13 (2.11–2.14)	2.0 (1.9–2.0)	2.32 (2.27–2.37)
		Denmark	1.2 (1.1–1.2)	1.15 (1.07–1.23)	0.9 (0.7–1.2)	1.04 (0.79–1.35)
		Finland	1.0 (0.9–1.1)	1.00	0.9 (0.6–1.1)	1.00
		Norway	1.0 (1.0–1.1)	1.06 (0.98–1.15)	0.6 (0.4–0.8)	0.70 (0.48–0.98)
		Sweden	1.0 (0.9–1.1)	1.03 (0.96–1.09)	0.9 (0.7–1.1)	1.07 (0.86–1.31)
BW (grams)	≥500	Canada	1.6 (1.5–1.6)	1.57 (1.52–1.62)	1.4 (1.2–1.5)	1.53 (1.36–1.72)
		USA	2.3 (2.3–2.3)	2.32 (2.30–2.33)	2.3 (2.2–2.3)	2.56 (2.51–2.61)
		Denmark	1.2 (1.1–1.3)	1.18 (1.10–1.26)	1.0 (0.8–1.3)	1.12 (0.86–1.42)
		Finland	1.0 (0.9–1.1)	1.00	0.9 (0.7–1.2)	1.00
		Norway	1.1 (1.1–1.2)	1.13 (1.05–1.22)	0.7 (0.5–0.9)	0.77 (0.55–1.05)
		Sweden	1.1 (1.0–1.2)	1.10 (1.04–1.16)	1.0 (0.9–1.3)	1.18 (0.97–1.43)
	≥1000	Canada	1.5 (1.4–1.5)	1.51 (1.46–1.57)	1.2 (1.0–1.3)	1.52 (1.33–1.72)
		USA	2.0 (2.0–2.1)	2.13 (2.12–2.14)	1.9 (1.9–2.0)	2.48 (2.43–2.54)
		Denmark	1.1 (1.1–1.2)	1.20 (1.12–1.28)	0.9 (0.7–1.2)	1.00 (0.76–1.30)
		Finland	1.0 (0.9–1.0)	1.00	0.8 (0.6–1.0)	1.00
		Norway	1.0 (1.0–1.1)	1.09 (1.01–1.18)	0.6 (0.4–0.8)	0.76 (0.53–1.06)
		Sweden	1.0 (0.9–1.1)	1.04 (0.98–1.11)	0.9 (0.7–1.1)	1.16 (0.93–1.42)
All live births		Canada	1.6 (1.5–1.6)	1.57 (1.52–1.62)	1.4 (1.2–1.5)	1.54 (1.36–1.73)
		USA	2.4 (2.3–2.4)	2.34 (2.32–2.35)	2.3 (2.3–2.3)	2.60 (2.54–2.65)
		Denmark	1.2 (1.1–1.3)	1.17 (1.09–1.25)	1.0 (0.8–1.3)	1.12 (0.86–1.43)
		Finland	1.0 (0.9–1.1)	1.00	0.9 (0.7–1.2)	1.00
		Norway	1.1 (1.1–1.2)	1.14 (1.06–1.22)	0.7 (0.5–1.0)	0.79 (0.56–1.08)
		Sweden	1.1 (1.0–1.2)	1.10 (1.04–1.17)	1.1 (0.9–1.3)	1.19 (0.98–1.44)

*GA* gestational age and *BW* birth weight. Rates are expressed per 1,000 infants at risk. Only births with gestational age <22 weeks excluded from the rate calculation for births **≥**22 weeks i.e., births with unknown gestational age included in the calculations. Calculations of rates for births **≥**28 weeks and birth weight **≥**500 g and **≥**1000 g were carried out in a similar manner. Finland was used as the reference category for calculation of rate ratios

Analyses of gestational age—and birth weight-specific perinatal and feto-infant mortality showed similar results (Appendix 2 Table 8 and Appendix 3 Table 9). Comparisons of cause-specific infant mortality rates for the period of 1995–2005 showed that infant deaths due to congenital anomalies, immaturity and other causes were all higher in Canada and the United States compared with the Nordic countries (except for Denmark, Appendix Table 4).

## Discussion

The higher rates of live births <22 and 22–23 weeks gestation in Canada as compared with the Nordic countries, and the lack of similar differences between them in live birth frequency at 24–25, 26–27 and 28–31 weeks and at 750–999 g, 1000–1499 g and 1500–1999 g suggests that the former differences may reflect dissimilarities in birth registration [18–20]. The observed differences in stillbirth and neonatal mortality may be a consequence of artifactual differences in birth registration. The unexpectedly low neonatal mortality rate among live births at 22–23 weeks gestation in Sweden may suggest the possibility of selective birth registration of survivors. The lower stillbirth:live birth ratios in the United States at 22–23 weeks and other preterm gestations may reflect a tendency to label non-viable live births as stillbirths. Between-country differences in crude versus gestational age-specific stillbirth and neonatal mortality rates may also stem from differences in birth registration. This is exemplified by the stillbirth contrast between Norway and Finland. Norway (which registered stillbirths from 16 week gestation) had substantially higher crude stillbirth rates compared with Finland (stillbirth registration criteria ≥22 weeks or ≥500 g), whereas no significant difference was observed after restriction to births ≥500 g, ≥1,000 g or ≥28 weeks. Finally, the lack of difference in crude versus gestational age—and birth weight-specific comparisons of post-neonatal mortality probably reflect the fact that near-viable births do not contribute substantially to post-neonatal deaths. The higher rates of post-neonatal mortality in Canada and the United States can be best explained by differences in factors such as the level of community-based social supports, preventative medical follow-up, strict surveillance for vaccination etc.

Our findings may indicate true differences in perinatal and postnatal health status between countries. We are not aware of any reports that non-viable live births are registered as stillbirths or labeled as miscarriages in the Nordic countries. In fact, a tendency to register live births <500 g as stillbirths has been reported from a few U.S. states [21]. The high rate of preterm birth in the United States relative to the Nordic countries is congruent with true differences in determinants of preterm birth such as body mass index, smoking, teen pregnancy and socioeconomic status. These factors could also explain higher live birth rates at very early gestation in the United States [22, 23].

Other explanations for our findings include between-country differences in stillbirth definitions including the exclusion of fetal deaths following pregnancy termination from stillbirth counts [24, 25] (Table 1). Finally, methods of estimation of gestational age vary between countries and need to be considered when comparing mortality rates. While ultrasound is considered the optimal method, combinations of last menstrual period, clinical estimates and ultrasound are often used.. These between-country differences in gestational age ascertainment may underlie some observed differences in mortality [14, 26]. For instance, misclassification due to the primary use of menstrual dating in the United States data may explain the slightly lower neonatal mortality rates at 22–23 weeks. The routine use of early ultrasound dating in Sweden, however, cannot explain the unexpectedly low neonatal mortality at 22–23 weeks.Our study has several limitations. We could not use data beyond 2005 since Canadian birth-death linked data were unavailable beyond 2005. Large datasets such as the ones we used are likely to contain errors. Systematic reporting differences can occur even within countries: for 2005, the CDC reported a neonatal mortality rate of 4.54 per 1000 live births based on all live births without any exclusion [27], while our estimated rate of 4.2 for the same year was based on live births with ≥20 completed weeks’ gestation. Errors in gestational age estimation are more likely at extremely low gestation. Correction of such errors differs by country. Some countries attempt to improve data quality by excluding very early gestation births with birth weight-for-gestational-age (z-score) above a certain threshold; infant mortality rates may change depending on whether or not such data checks are carried out, whether the gestational age of such births is set to missing and whether or not such births are deleted from vital records.

## Conclusions

Our study shows substantial international variation in the frequency and mortality of near-viable births. Reasons to suspect that the observed variations are artifacts of birth registration differences include similar live birth frequencies in other very preterm/low birth weight categories, some implausibly low neonatal mortality rates at the borderline of viability, large between-country differences in stillbirth ratios at very preterm gestation, and differences in between-country contrasts of crude versus gestational age—and birth weight-specific stillbirths and neonatal mortality. On the other hand, true between-country differences in maternal, fetal and infant health status could also explain the observed findings; particularly for post-neonatal mortality. The distribution of risk factors for preterm birth in different countries and the high rates of preterm birth in the United States relative to the Nordic countries are congruent with the observed higher rates of live births at the borderline of viability in the United States. Also, discrepancies in birth registration (labeling nonviable live births as stillbirths) have been reported from the United States but not from the Nordic countries. Our study cannot provide a definitive explanation for the observed differences in the frequency and mortality of live births at the borderline of viability and at very early gestation. More detailed studies are required to assess the value of international comparisons of fetal and infant mortality and to make such comparisons more meaningful.

## Appendix 1

**Table 7:** Crude stillbirth rates and rate ratios, and stillbirth rates and rate ratios after excluding live births and stillbirths <22 weeks and <28 weeks, among selected high-income countries, 1995-2005 and 2005

Gestational age/Birth weight			1995–2005		2005	
			Rate (95 % CI)	Rate Ratio (95 % CI)	Rate (95 % CI)	Rate Ratio (95 % CI)
GA (weeks)	≥22	Canada	4.8 (4.7–4.9)	1.27 (1.24–1.29)	5.0 (4.7–5.3)	1.55 (1.45–1.64)
		USA	5.7 (5.7–5.8)	1.50 (1.49–1.51)	5.4 (5.4–5.5)	1.70 (1.68–1.72)
		Denmark^a^	4.4 (4.2–4.5)	1.14 (1.10–1.18)	4.8 (4.3–5.4)	1.51 (1.35–1.69)
		Finland	3.8 (3.7–4.0)	1.00	3.2 (2.8–3.7)	1.00
		Iceland	4.3 (3.7–4.9)	1.12 (0.97–1.29)	4.9 (3.0–7.4)	1.52 (0.94–2.33)
		Norway	5.0 (4.8–5.2)	1.31 (1.26–1.35)	3.7 (3.2–4.3)	1.17 (1.01–1.33)
		Sweden^a^	3.4 (3.3–3.6)	0.90 (0.87–0.93)	2.9 (2.6–3.3)	0.92 (0.81–1.03)
	≥28	Canada	3.2 (3.1–3.2)	0.98 (0.96–1.00)	3.0 (2.7–3.2)	1.13 (1.05–1.23)
		USA	3.8 (3.8–3.8)	1.17 (1.16–1.18)	3.5 (3.5–3.6)	1.34 (1.32–1.36)
		Denmark	4.1 (4.0–4.3)	1.29 (1.24–1.33)	3.4 (3.0–3.9)	1.32 (1.15–1.50)
		Finland	3.2 (3.1–3.4)	1.00	2.6 (2.2–3.1)	1.00
		Iceland	2.8 (2.3–3.3)	0.85 (0.71–1.02)	1.4 (0.5–3.0)	0.53 (0.20–1.16)
		Norway	3.5 (3.3–3.6)	1.07 (1.03–1.12)	2.5 (2.1–2.9)	0.95 (0.80–1.12)
		Sweden	3.5 (3.3–3.6)	1.07 (1.04–1.11)	2.9 (2.6–3.3)	1.12 (1.00–1.26)
BW (grams)	≥500	Canada	4.6 (4.5–4.6)	1.37 (1.34–1.39)	4.4 (4.1–4.7)	1.60 (1.49–1.70)
		USA	5.1 (5.1–5.1)	1.53 (1.52–1.53)	4.8 (4.7–4.9)	1.74 (1.71–1.76)
		Denmark	4.3 (4.1–4.4)	1.28 (1.23–1.32)	4.4 (3.9–4.9)	1.58 (1.40–1.77)
		Finland	3.3 (3.2–3.5)	1.00	2.8 (2.4–3.2)	1.00
		Iceland	3.8 (3.2–4.4)	1.14 (0.98–1.32)	3.5 (1.9–5.7)	1.26 (0.70–2.07)
		Norway	4.2 (4.0–4.3)	1.26 (1.21–1.30)	3.1 (2.6–3.6)	1.11 (0.96–1.29)
		Sweden	3.5 (3.4–3.6)	1.04 (1.01–1.08)	2.9 (2.6–3.2)	1.04 (0.92–1.17)
	≥1000	Canada	3.2 (3.1–3.3)	1.29 (1.26–1.32)	2.9 (2.7–3.1)	1.36 (1.25–1.47)
		USA	3.8 (3.8–3.8)	1.54 (1.53–1.55)	3.6 (3.6–3.7)	1.71 (1.68–1.74)
		Denmark	3.9 (3.7–4.0)	1.57 (1.51–1.62)	3.5 (3.0–4.0)	1.65 (1.44–1.88)
		Finland	2.5 (2.4–2.6)	1.00	2.1 (1.8–2.5)	1.00
		Iceland	2.5 (2.1–3.0)	1.01 (0.83–1.21)	1.4 (0.5–3.0)	0.66 (0.24–1.44)
		Norway	3.1 (3.0–3.3)	1.25 (1.20–1.31)	2.4 (2.0–2.8)	1.12 (0.94–1.33)
		Sweden	3.2 (3.1–3.4)	1.31 (1.26–1.35)	2.7 (2.4–3.1)	1.28 (1.14–1.44)
All births		Canada	5.8 (5.7–5.9)	1.52 (1.49–1.55)	6.2 (5.9–6.6)	1.93 (1.83–2.04)
		USA	7.0 (6.9–7.0)	1.81 (1.81–1.82)	6.7 (6.6–6.8)	2.08 (2.06–2.11)
		Denmark	4.4 (4.2–4.5)	1.14 (1.10–1.18)	4.8 (4.3–5.4)	1.50 (1.34–1.68)
		Finland	3.8 (3.7–4.0)	1.00	3.2 (2.8–3.7)	1.00
		Iceland	4.3 (3.7–4.9)	1.11 (0.97–1.28)	4.9 (3.0–7.4)	1.51 (0.94–2.31)
		Norway	8.9 (8.7–9.1)	2.32 (2.26–2.38)	7.9 (7.2–8.6)	2.45 (2.23–2.69)
		Sweden	3.5 (3.4–3.6)	0.91 (0.88–0.94)	2.9 (2.6–3.3)	0.91 (0.81–1.02)

Rates are expressed per 1,000 total births

^a^Denmark and Sweden did not report stillbirths less than 28 weeks until 2004 and 2008, respectively

Only births with gestational age <22 weeks excluded from the rate calculation for births **≥**22 weeks i.e., births with unknown gestational age included in the calculations. Calculations of rates for births **≥**28 weeks and birth weight **≥**500 g and **≥**1000 g were carried out in a similar manner. Finland was used as the reference category for calculation of rate ratios

## Appendix 2

**Table 8:** Perinatal mortality rates and rate ratios by gestational age (GA) and birth weight (BW) among selected high-income countries, 1995–2005 and 2005

Gestational age/Birth weight			1995–2005		2005	
			Rate ( 95 % CI)	Rate Ratio ( 95 % CI)	Rate ( 95 % CI)	Rate Ratio ( 95 % CI)
GA (weeks)	≥22	Canada	8.0 (7.9–8.1)	1.26 (1.24–1.28)	8.1 (7.7–8.5)	1.52 (1.45–1.60)
		USA	9.5 (9.4–9.5)	1.50 (1.50–1.50)	9.0 (8.9–9.1)	1.68 (1.67–1.70)
		Denmark^a^	7.7 (7.5–7.9)	1.23 (1.19–1.26)	8.1 (7.4–8.8)	1.51 (1.38–1.64)
		Finland	6.3 (6.1–6.5)	1.00	5.3 (4.8–6.0)	1.00
		Iceland	6.3 (5.6–7.1)	1.01 (0.89–1.13)	6.3 (4.1–9.1)	1.17 (0.77–1.71)
		Norway	7.4 (7.2–7.6)	1.17 (1.14–1.21)	5.6 (5.0–6.2)	1.05 (0.94–1.17)
		Sweden^a^	5.7 (5.5–5.8)	0.90 (0.88–0.92)	4.4 (4.0–4.8)	0.82 (0.75–0.90)
	≥28	Canada	4.8 (4.7–4.9)	0.92 (0.90–0.94)	4.5 (4.2–4.8)	1.02 (0.95–1.08)
		USA	5.6 (5.6–5.6)	1.07 (1.07–1.08)	5.2 (5.1–5.2)	1.16 (1.14–1.17)
		Denmark	6.6 (6.5–6.8)	1.27 (1.23–1.30)	5.7 (5.1–6.3)	1.27 (1.15–1.41)
		Finland	5.2 (5.1–5.4)	1.00	4.5 (3.9–5.0)	1.00
		Iceland	3.8 (3.3–4.4)	0.73 (0.62–0.84)	1.9 (0.8–3.7)	0.42 (0.18–0.83)
		Norway	5.0 (4.9–5.2)	0.96 (0.93–1.00)	3.7 (3.2–4.2)	0.83 (0.72–0.95)
		Sweden	5.0 (4.9–5.1)	0.96 (0.93–0.98)	3.9 (3.5–4.3)	0.88 (0.79–0.97)
BW (grams)	≥500	Canada	7.4 (7.3–7.5)	1.31 (1.29–1.33)	7.2 (6.8–7.5)	1.51 (1.44–1.59)
		USA	8.3 (8.3–8.4)	1.48 (1.48–1.49)	7.8 (7.7–7.9)	1.65 (1.63–1.67)
		Denmark	7.5 (7.3–7.7)	1.33 (1.30–1.37)	7.4 (6.8–8.1)	1.57 (1.43–1.72)
		Finland	5.6 (5.4–5.8)	1.00	4.7 (4.2–5.3)	1.00
		Iceland	5.8 (5.1–6.5)	1.03 (0.91–1.16)	4.6 (2.8–7.2)	0.98 (0.60–1.52)
		Norway	6.4 (6.2–6.6)	1.14 (1.10–1.17)	4.8 (4.3–5.4)	1.01 (0.90–1.14)
		Sweden	5.6 (5.5–5.7)	0.99 (0.97–1.02)	4.3 (3.9–4.7)	0.90 (0.82–0.99)
	≥1000	Canada	4.9 (4.8–4.9)	1.22 (1.19–1.24)	4.3 (4.0–4.6)	1.24 (1.16–1.33)
		USA	5.6 (5.6–5.6)	1.40 (1.39–1.40)	5.2 (5.2–5.3)	1.51 (1.49–1.53)
		Denmark	6.4 (6.2–6.5)	1.59 (1.55–1.64)	5.7 (5.2–6.4)	1.66 (1.49–1.83)
		Finland	4.0 (3.8–4.1)	1.00	3.5 (3.0–4.0)	1.00
		Iceland	3.4 (2.9–4.0)	0.86 (0.73–1.01)	1.9 (0.8–3.7)	0.54 (0.23–1.06)
		Norway	4.6 (4.5–4.8)	1.16 (1.12–1.20)	3.6 (3.1–4.2)	1.04 (0.91–1.20)
		Sweden	4.8 (4.7–5.0)	1.22 (1.18–1.25)	3.7 (3.4–4.1)	1.08 (0.97–1.20)
All births		Canada	9.6 (9.4–9.7)	1.51 (1.49–1.53)	10.1 (9.7–10.6)	1.89 (1.81–1.97)
		USA	11.3 (11.3–11.4)	1.79 (1.78–1.79)	10.9 (10.8–11.0)	2.03 (2.01–2.05)
		Denmark	7.8 (7.6–8.0)	1.23 (1.20–1.26)	8.1 (7.5–8.9)	1.52 (1.39–1.65)
		Finland	6.3 (6.1–6.5)	1.00	5.4 (4.8–6.0)	1.00
		Iceland	6.4 (5.7–7.2)	1.01 (0.90–1.13)	6.3 (4.1–9.1)	1.17 (0.77–1.70)
		Norway	11.5 (11.3–11.8)	1.82 (1.78–1.86)	9.9 (9.1–10.8)	1.85 (1.70–2.01)
		Sweden	5.7 (5.6–5.9)	0.90 (0.88–0.93)	4.4 (4.0–4.8)	0.81 (0.74–0.89)

Rates are expressed per 1,000 total births. Numerator = Stillbirths + Neonatal deaths

^a^Denmark and Sweden did not report on stillbirths less than 28 weeks until 2004 and 2008 respectively

Finland was used as the reference category for calculation of rate ratios

Only births with gestational age <22 weeks excluded from the rate calculation for births **≥**22 weeks i.e., births with unknown gestational age included in the calculations. Calculations of rates for births **≥**28 weeks and birth weight **≥**500 g and **≥**1000 g were carried out in a similar manner

## Appendix 3

**Table 9:** Feto-infant mortality rates and rate ratios by gestational age (GA) and birth weight (BW) among selected high-income countries, 1995–2005 and 2005

Gestational age/Birth weight			1995–2005		2005	
			Rate (95 % CI)	Rate Ratio (95 % CI)	Rate (95 % CI)	Rate Ratio (95 % CI)
GA (weeks)	≥22	Canada	9.5 (9.4–9.7)	1.30 (1.29–1.32)	9.5 (9.0–9.9)	1.52 (1.46–1.59)
		USA	11.8 (11.8–11.8)	1.61 (1.61–1.62)	11.2 (11.1–11.3)	1.81 (1.79–1.83)
		Denmark^a^	8.9 (8.7–9.1)	1.22 (1.19–1.25)	9.1 (8.3–9.8)	1.45 (1.34–1.58)
		Finland	7.3 (7.1–7.5)	1.00	6.2 (5.6–6.9)	1.00
		Norway	8.5 (8.3–8.7)	1.16 (1.13–1.19)	6.3 (5.6–7.0)	1.01 (0.91–1.12)
		Sweden^a^	6.8 (6.6–6.9)	0.93 (0.91–0.95)	5.4 (5.0–5.9)	0.87 (0.80–0.95)
	≥28	Canada	6.3 (6.2–6.4)	1.01 (0.99–1.02)	5.7 (5.4–6.1)	1.09 (1.03–1.15)
		USA	7.7 (7.7–7.7)	1.24 (1.23–1.24)	7.1 (7.0–7.2)	1.35 (1.33–1.36)
		Denmark	7.8 (7.6–8.0)	1.25 (1.22–1.28)	6.6 (5.9–7.2)	1.24 (1.12–1.36)
		Finland	6.2 (6.0–6.4)	1.00	5.3 (4.7–5.9)	1.00
		Norway	6.0 (5.8–6.2)	0.97 (0.94–1.00)	4.3 (3.8–4.9)	0.81 (0.71–0.92)
		Sweden	6.0 (5.9–6.2)	0.97 (0.94–0.99)	4.8 (4.4–5.3)	0.91 (0.83–0.99)
BW (grams)	≥500	Canada	8.9 (8.8–9.1)	1.34 (1.33–1.37)	8.5 (8.1–8.9)	1.52 (1.45–1.59)
		USA	10.6 (10.6–10.7)	1.61 (1.60–1.61)	10.1 (10.0–10.2)	1.79 (1.78–1.81)
		Denmark	8.7 (8.5–8.9)	1.31 (1.28–1.34)	8.4 (7.7–9.2)	1.50 (1.38–1.63)
		Finland	6.6 (6.4–6.8)	1.00	5.6 (5.0–6.3)	1.00
		Norway	7.5 (7.3–7.7)	1.13 (1.10–1.17)	5.5 (4.9–6.1)	0.98 (0.87–1.09)
		Sweden	6.7 (6.5–6.8)	1.01 (1.00–1.05)	5.3 (4.9–5.8)	0.95 (0.87–1.03)
	≥1000	Canada	6.3 (6.2–6.4)	1.27 (1.26–1.30)	5.5 (5.2–5.8)	1.29 (1.21–1.36)
		USA	7.6 (7.6–7.6)	1.55 (1.54–1.55)	7.2 (7.1–7.2)	1.68 (1.66–1.70)
		Denmark	7.5 (7.3–7.7)	1.52 (1.48–1.56)	6.6 (6.0–7.3)	1.56 (1.42–1.71)
		Finland	4.9 (4.8–5.1)	1.00	4.3 (3.7–4.8)	1.00
		Norway	5.7 (5.5–5.8)	1.14 (1.11–1.18)	4.2 (3.7–4.8)	0.99 (0.87–1.13)
		Sweden	5.8 (5.7–6.0)	1.19 (1.16–1.22)	4.6 (4.2–5.1)	1.09 (0.99–1.19)
All births		Canada	11.1 (11.0–11.3)	1.51 (1.49–1.53)	11.5 (11.0–11.9)	1.84 (1.77–1.92)
		USA	13.7 (13.6–13.7)	1.86 (1.85–1.86)	13.2 (13.1–13.3)	2.12 (2.10–2.13)
		Denmark	9.0 (8.8–9.2)	1.22 (1.19–1.25)	9.1 (8.4–9.9)	1.46 (1.35–1.59)
		Finland	7.4 (7.2–7.6)	1.00	6.2 (5.6–6.9)	1.00
		Norway	12.7 (12.4–12.9)	1.72 (1.68–1.76)	10.6 (9.8–11.5)	1.71 (1.57–1.85)
		Sweden	6.8 (6.7–7.0)	0.93 (0.90–0.95)	5.4 (5.0–5.9)	0.87 (0.80–0.95)

Rates are expressed per 1,000 total births. Numerator = Stillbirths + Infant deaths

^a^Denmark and Sweden did not report on stillbirths less than 28 weeks until 2004 and 2008, respectively

Only births with gestational age <22 weeks excluded from the rate calculation for births ≥22 weeks i.e., births with unknown gestational age included in the calculations

Calculations of rates for births ≥28 weeks and birth weight ≥500 g and ≥1000 g were carried out in a similar manner

Finland was used as the reference category for calculation of rate ratios

## Appendix 4

**Table 10:** Cause-specific infant mortality rates among selected high-income countries, 1995–2005

Gestational age (weeks)/Birth weight (grams)		Canada			USA			Denmark			Finland			Norway^a^			Sweden		
		CA	Imm-rel	Other	CA	Imm-rel	Other	CA	Imm-rel	Other	CA	Imm-rel	Other	CA	Imm-rel	Other	CA	Imm-rel	Other
GA	≥22	1.32	1.12	2.19	1.40	1.66	2.93	1.41	1.07	1.54	1.27	0.98	1.30	1.11	0.39	1.92	1.16	0.51	1.62
	≥28	1.16	0.20	1.65	1.27	0.27	2.25	1.37	0.32	1.37	1.24	0.20	1.07	1.08	0.11	1.32	1.11	0.13	1.27
	≥32	1.04	0.10	1.52	1.14	0.14	2.04	1.29	0.16	1.30	1.14	0.09	0.98	0.99	0.05	1.15	1.00	0.06	1.12
	≥37	0.72	0.06	1.30	0.80	0.08	1.73	1.05	0.10	1.13	0.81	0.04	0.82	0.69	0.04	1.00	0.67	0.03	0.93
BW	≥500	1.30	0.94	2.07	1.39	1.32	2.82	1.42	0.96	1.48	1.26	0.82	1.24	1.09	0.33	1.81	1.04	0.42	1.48
	≥1,000	1.15	0.19	1.67	1.26	0.25	2.24	1.37	0.30	1.34	1.21	0.18	1.06	1.04	0.11	1.32	0.98	0.12	1.21
	≥1,500	1.03	0.11	1.54	1.11	0.15	2.09	1.24	0.13	1.26	1.09	0.10	1.00	0.93	0.06	1.18	0.87	0.07	1.08
All live births		1.42	1.41	2.40	1.45	2.14	3.11	1.42	1.11	1.53	1.26	0.98	1.28	1.10	0.41	1.99	1.04	0.49	1.53

Rates are expressed per 1,000 live births

CA denotes congenital anomalies and Imm-rel denotes immaturity-related causes of infant death

^**a**^Norway data for all BW and GA categories is from 1996–2005

All calculations exclude live births with unknown birth weight and unknown gestational age

ICD-10 codes used for CA: Q00-Q99

ICD-10 codes used for Immaturity-related conditions: D58.9, P01.3–P01.5, P01.8–P01.9, P02.7, P05.0–P05.9, P07.0–P07.3, P10.2, P22.0–P22.9, P25.0–P25.8, P26.0–P26.9, P27.0–P27.9, P28.0–P28.9, P29.0-29.2, P29.4–P29.9, P52.0–P52.3, P57.8–P57.9, P58.0-P58.9, P59.0–P59.9, P77, P78.0, P80.0, P91.1–P91.2, P91.8, P94.1–P94.9, P96.0, P96.3–P96.5

ICD-10 codes used for other conditions: rest of A000 - Y899

## Additional file

Additional file 1:
**STROBE Statement—checklist of items that should be included in reports of observational studies.** (DOC 84 kb)

## Abbreviations

(GA): Gestational age
(BW): Birth weight
